# 4-Chloro-2-methyl-*N*-phenyl­benzene­sulfonamide

**DOI:** 10.1107/S1600536809003845

**Published:** 2009-02-06

**Authors:** B. Thimme Gowda, Sabine Foro, P. G. Nirmala, K. S. Babitha, Hartmut Fuess

**Affiliations:** a Department of Chemistry, Mangalore University, Mangalagangotri 574 199, Mangalore, India; b Institute of Materials Science, Darmstadt University of Technology, Petersenstrasse 23, D-64287 Darmstadt, Germany

## Abstract

There are two mol­ecules in the asymmetric unit of the title compound, C_13_H_12_ClNO_2_S, with similar conformations. The orientations of the *ortho*-methyl groups in the sulfonyl benzene rings are in the direction of the N—H bonds of the sulfonamide groups. In the crystal, the mol­ecules are each linked into centrosymmetric dimers through N—H⋯O hydrogen bonds and packed into a layered structure diagonally in the *bc* plane.

## Related literature

For related structures, see: Gelbrich *et al.* (2007 ▶); Gowda *et al.* (2008**a* ▶,b* ▶, 2009 ▶); Perlovich *et al.* (2006 ▶)
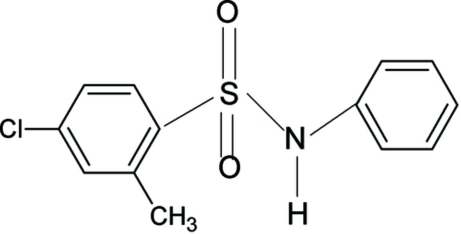


## Experimental

### 

#### Crystal data

C_13_H_12_ClNO_2_S
*M* *_r_* = 281.75
Triclinic, 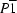

*a* = 8.609 (1) Å
*b* = 11.143 (1) Å
*c* = 14.726 (2) Å
α = 98.618 (7)°
β = 90.951 (8)°
γ = 105.79 (1)°
*V* = 1341.6 (3) Å^3^
*Z* = 4
Cu *K*α radiation
μ = 3.93 mm^−1^
*T* = 299 (2) K
0.33 × 0.23 × 0.08 mm

#### Data collection

Enraf–Nonius CAD-4 diffractometer
Absorption correction: ψ scan (North *et al.*, 1968 ▶) *T*_min_ = 0.350, *T*_max_ = 0.729
7620 measured reflections
4784 independent reflections
2980 reflections with *I* > 2σ(*I*)
*R*_int_ = 0.045
3 standard reflections frequency: 120 min intensity decay: 1.0%

#### Refinement

*R*[*F* ^2^ > 2σ(*F* ^2^)] = 0.057
*wR*(*F* ^2^) = 0.176
*S* = 1.03
4784 reflections
333 parameters
12 restraints
H atoms treated by a mixture of independent and constrained refinement
Δρ_max_ = 0.41 e Å^−3^
Δρ_min_ = −0.42 e Å^−3^

### 

Data collection: *CAD-4-PC* (Enraf–Nonius, 1996 ▶); cell refinement: *CAD-4-PC*; data reduction: *REDU4* (Stoe & Cie, 1987 ▶); program(s) used to solve structure: *SHELXS97* (Sheldrick, 2008 ▶); program(s) used to refine structure: *SHELXL97* (Sheldrick, 2008 ▶); molecular graphics: *PLATON* (Spek, 2003 ▶); software used to prepare material for publication: *SHELXL97*.

## Supplementary Material

Crystal structure: contains datablocks I, global. DOI: 10.1107/S1600536809003845/fj2192sup1.cif

Structure factors: contains datablocks I. DOI: 10.1107/S1600536809003845/fj2192Isup2.hkl

Additional supplementary materials:  crystallographic information; 3D view; checkCIF report

## Figures and Tables

**Table 1:** Hydrogen-bond geometry (Å, °)

*D*—H⋯*A*	*D*—H	H⋯*A*	*D*⋯*A*	*D*—H⋯*A*
N1—H1*N*⋯O2^i^	0.91 (5)	2.02 (5)	2.922 (4)	175 (4)
N2—H2*N*⋯O3^ii^	0.88 (4)	2.03 (5)	2.906 (4)	173 (4)

Symmetry codes: (i) 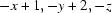; (ii) 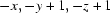.

**Fig. 1.**
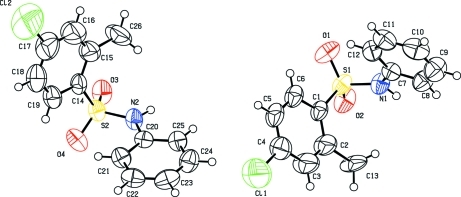
Molecular structure of the title compound, showing the atom labeling scheme. The displacement ellipsoids are drawn at the 50% probability level. The H atoms are represented as small spheres of arbitrary radii.

**Fig. 2.**
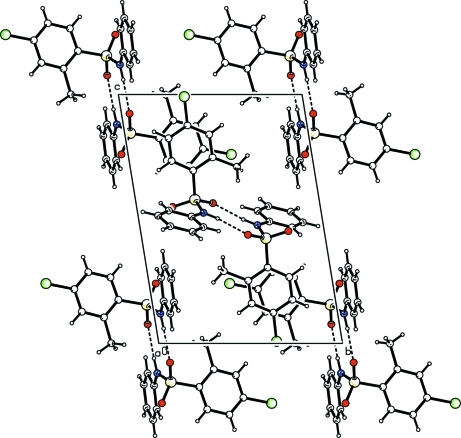
Molecular packing of the title compound with hydrogen bonding shown as dashed lines.

## supplementary crystallographic information

### Comment

In the present work, as part of a study of substituent effects on the structures of *N*-(aryl)-arylsulfonamides, the structure of *N*-(phenyl)-2-methyl-4-chlorobenzenesulfonamide (NP2M4CBSA) has been determined (Gowda *et al.* 2008*a,b*, 2009). The asymmetric unit of NP2M4CBSA contains 2 molecules. The orientations of the *ortho*- methyl groups in the sulfonyl benzene rings are in the direction of the N—H bonds of the sulfonamido groups (Fig. 1). The opposite signs of the C—S—N—C torsion angles in the two independent molecules, -61.9 (4)° (molecule 1) and 69.7 (4)° (molecule 2), indicates that they have opposite chirality, although the choice of the chirality of the second molecule relative to the first may be arbitary. The two benzene rings in NP2M4CBSA are tilted relative to each other by 86.6 (2)° in the molecule 1 and 83.0 (2)° in molecule 2, compared with the values of 67.5 (1)° (molecule 1) and 72.9 (1)° (molecule 2) for *N*-(phenyl)-2,4-dimethylbenzenesulfonamide (NP24DMBSA) (Gowda *et al.*, 2009). The other bond parameters in NP2M4CBSA are similar to those observed for *N*-(2-methylphenyl)-benzenesulfonamide (Gowda *et al.*, 2008*a*), NP24DMBSA and other aryl sulfonamides (Perlovich *et al.*, 2006; Gelbrich *et al.*, 2007; Gowda *et al.*, 2008*b*). The crystal packing of molecules in NP2M4CBSA *via* intermolecular N—H···O hydrogen bonds (Table 1) is shown in Fig.2.

### Experimental

The solution of *m*-chlorotoluene (10 cc) in chloroform (40 cc) was treated dropwise with chlorosulfonic acid (25 cc) at 0 ° C. After the initial evolution of hydrogen chloride subsided, the reaction mixture was brought to room temperature and poured into crushed ice in a beaker. The chloroform layer was separated, washed with cold water and allowed to evaporate slowly. The residual 2-methyl-4-chlorobenzenesulfonylchloride was treated with aniline in the stoichiometric ratio and boiled for ten minutes. The reaction mixture was then cooled to room temperature and added to ice cold water (100 cc). The resultant solid *N*-(phenyl)-2-methyl-4-chlorobenzenesulfonamide was filtered under suction and washed thoroughly with cold water. It was then recrystallized to constant melting point from dilute ethanol. The purity of the compound was checked and characterized by recording its infrared and NMR spectra. The single crystals used in X-ray diffraction studies were grown in ethanolic solution by slow evaporation at room temperature.

### Refinement

The H atoms of the NH groups were located in a diffrerence map and their positions refined, with N—H = 0.88 (4)–0.91 (5) Å. The carbon-bound H atoms were positioned with idealized geometry and refined using a riding model, with C—H distances 0.93–0.96 Å. All H atoms were refined with isotropic displacement parameters (set to 1.2 times of the *U*_eq_ of the parent atom). For methyl group *U*_iso_(H) = 1.5 *U*_eq_.

### Crystal data

**Table:**

C_13_H_12_ClNO_2_S	*Z* = 4
*M**_r_* = 281.75	*F*(000) = 584
Triclinic, *P*1	*D*_x_ = 1.395 Mg m^−^^3^
Hall symbol: -P 1	Cu *K*α radiation, λ = 1.54180 Å
*a* = 8.609 (1) Å	Cell parameters from 25 reflections
*b* = 11.143 (1) Å	θ = 5.6–21.8°
*c* = 14.726 (2) Å	µ = 3.93 mm^−^^1^
α = 98.618 (7)°	*T* = 299 K
β = 90.951 (8)°	Prism, colourless
γ = 105.79 (1)°	0.33 × 0.23 × 0.08 mm
*V* = 1341.6 (3) Å^3^	

### Data collection

**Table:**

Enraf–Nonius CAD-4 diffractometer	2980 reflections with *I* > 2σ(*I*)
Radiation source: fine-focus sealed tube	*R*_int_ = 0.045
graphite	θ_max_ = 67.0°, θ_min_ = 3.0°
ω/2θ scans	*h* = −10→10
Absorption correction: ψ scan (North *et al.*, 1968)	*k* = −13→13
*T*_min_ = 0.350, *T*_max_ = 0.729	*l* = −17→7
7620 measured reflections	3 standard reflections every 120 min
4784 independent reflections	intensity decay: 1.0%

### Refinement

**Table:**

Refinement on *F*^2^	Primary atom site location: structure-invariant direct methods
Least-squares matrix: full	Secondary atom site location: difference Fourier map
*R*[*F*^2^ > 2σ(*F*^2^)] = 0.057	Hydrogen site location: inferred from neighbouring sites
*wR*(*F*^2^) = 0.176	H atoms treated by a mixture of independent and constrained refinement
*S* = 1.03	*w* = 1/[σ^2^(*F*_o_^2^) + (0.0962*P*)^2^ + 0.1233*P*] where *P* = (*F*_o_^2^ + 2*F*_c_^2^)/3
4784 reflections	(Δ/σ)_max_ = 0.005
333 parameters	Δρ_max_ = 0.41 e Å^−^^3^
12 restraints	Δρ_min_ = −0.42 e Å^−^^3^

### Special details

**Table:**

Geometry. All e.s.d.'s (except the e.s.d. in the dihedral angle between two l.s. planes) are estimated using the full covariance matrix. The cell e.s.d.'s are taken into account individually in the estimation of e.s.d.'s in distances, angles and torsion angles; correlations between e.s.d.'s in cell parameters are only used when they are defined by crystal symmetry. An approximate (isotropic) treatment of cell e.s.d.'s is used for estimating e.s.d.'s involving l.s. planes.
Refinement. Refinement of *F*^2^ against ALL reflections. The weighted *R*-factor *wR* and goodness of fit *S* are based on *F*^2^, conventional *R*-factors *R* are based on *F*, with *F* set to zero for negative *F*^2^. The threshold expression of *F*^2^ > σ(*F*^2^) is used only for calculating *R*-factors(gt) *etc*. and is not relevant to the choice of reflections for refinement. *R*-factors based on *F*^2^ are statistically about twice as large as those based on *F*, and *R*- factors based on ALL data will be even larger.

### Fractional atomic coordinates and isotropic or equivalent isotropic displacement parameters (Å^2^)

**Table:**

	*x*	*y*	*z*	*U*_iso_*/*U*_eq_	
Cl1	0.4618 (4)	0.43860 (18)	0.24063 (15)	0.1670 (10)	
S1	0.46343 (11)	0.96896 (11)	0.15504 (7)	0.0586 (3)	
O1	0.4383 (4)	1.0364 (3)	0.2403 (2)	0.0802 (9)	
O2	0.3532 (3)	0.9562 (3)	0.07717 (19)	0.0722 (8)	
N1	0.6377 (4)	1.0345 (3)	0.1199 (2)	0.0611 (9)	
H1N	0.647 (5)	1.039 (4)	0.059 (3)	0.073*	
C1	0.4664 (4)	0.8182 (4)	0.1749 (3)	0.0565 (10)	
C2	0.4758 (8)	0.7240 (5)	0.1044 (3)	0.0907 (16)	
C3	0.4778 (10)	0.6088 (5)	0.1273 (4)	0.120 (2)	
H3	0.4876	0.5444	0.0816	0.144*	
C4	0.4653 (7)	0.5877 (5)	0.2179 (4)	0.0910 (16)	
C5	0.4560 (6)	0.6791 (5)	0.2862 (3)	0.0768 (13)	
H5	0.4494	0.6642	0.3467	0.092*	
C6	0.4563 (5)	0.7946 (5)	0.2653 (3)	0.0663 (11)	
H6	0.4495	0.8586	0.3122	0.080*	
C7	0.7894 (4)	1.0635 (3)	0.1698 (3)	0.0522 (9)	
C8	0.9247 (5)	1.0883 (4)	0.1201 (3)	0.0701 (12)	
H8	0.9151	1.0848	0.0567	0.084*	
C9	1.0763 (6)	1.1187 (5)	0.1656 (4)	0.0865 (15)	
H9	1.1685	1.1358	0.1323	0.104*	
C10	1.0914 (6)	1.1239 (5)	0.2591 (4)	0.0834 (15)	
H10	1.1933	1.1440	0.2891	0.100*	
C11	0.9570 (6)	1.0995 (4)	0.3074 (3)	0.0718 (12)	
H11	0.9671	1.1026	0.3707	0.086*	
C12	0.8054 (5)	1.0701 (4)	0.2639 (3)	0.0653 (11)	
H12	0.7141	1.0547	0.2980	0.078*	
C13	0.4894 (9)	0.7419 (4)	0.0007 (3)	0.102 (2)	
H13A	0.5834	0.8097	−0.0050	0.123*	
H13B	0.3947	0.7616	−0.0205	0.123*	
H13C	0.4985	0.6653	−0.0357	0.123*	
Cl2	0.1160 (4)	0.3570 (3)	0.99174 (14)	0.1919 (12)	
S2	−0.06931 (11)	0.32374 (9)	0.57752 (8)	0.0560 (3)	
O3	−0.1468 (3)	0.4185 (3)	0.5635 (2)	0.0703 (8)	
O4	−0.1553 (3)	0.1953 (3)	0.5486 (2)	0.0727 (8)	
N2	0.0946 (4)	0.3586 (3)	0.5248 (2)	0.0581 (9)	
H2N	0.114 (5)	0.431 (4)	0.503 (3)	0.070*	
C14	−0.0126 (5)	0.3404 (4)	0.6948 (3)	0.0569 (10)	
C15	0.0592 (7)	0.4573 (4)	0.7478 (4)	0.0803 (13)	
C16	0.0983 (8)	0.4591 (6)	0.8395 (4)	0.115 (2)	
H16	0.1457	0.5359	0.8770	0.138*	
C17	0.0679 (8)	0.3489 (7)	0.8762 (4)	0.110 (2)	
C18	−0.0041 (7)	0.2348 (6)	0.8243 (4)	0.0906 (16)	
H18	−0.0267	0.1612	0.8502	0.109*	
C19	−0.0424 (5)	0.2309 (4)	0.7334 (3)	0.0679 (11)	
H19	−0.0894	0.1533	0.6967	0.081*	
C20	0.2114 (4)	0.2902 (3)	0.5100 (2)	0.0477 (8)	
C21	0.1911 (5)	0.1719 (4)	0.5338 (3)	0.0610 (10)	
H21	0.0998	0.1346	0.5632	0.073*	
C22	0.3085 (6)	0.1093 (4)	0.5135 (3)	0.0692 (12)	
H22	0.2954	0.0292	0.5286	0.083*	
C23	0.4443 (6)	0.1657 (5)	0.4708 (3)	0.0742 (13)	
H23	0.5231	0.1238	0.4574	0.089*	
C24	0.4630 (5)	0.2827 (5)	0.4484 (3)	0.0723 (12)	
H24	0.5551	0.3209	0.4201	0.087*	
C25	0.3464 (4)	0.3451 (4)	0.4674 (3)	0.0577 (10)	
H25	0.3596	0.4247	0.4513	0.069*	
C26	0.0961 (9)	0.5821 (4)	0.7100 (4)	0.111 (2)	
H26A	0.1530	0.5749	0.6548	0.133*	
H26B	0.1619	0.6486	0.7550	0.133*	
H26C	−0.0033	0.6010	0.6965	0.133*	

### Atomic displacement parameters (Å^2^)

**Table:**

	*U*^11^	*U*^22^	*U*^33^	*U*^12^	*U*^13^	*U*^23^
Cl1	0.280 (3)	0.1079 (13)	0.1210 (15)	0.0456 (15)	0.0296 (16)	0.0575 (11)
S1	0.0529 (5)	0.0868 (7)	0.0417 (5)	0.0282 (5)	0.0004 (4)	0.0114 (5)
O1	0.092 (2)	0.114 (3)	0.0494 (17)	0.0576 (19)	0.0072 (15)	0.0056 (16)
O2	0.0544 (16)	0.121 (3)	0.0516 (17)	0.0361 (16)	−0.0077 (13)	0.0244 (16)
N1	0.0573 (19)	0.085 (2)	0.0421 (18)	0.0178 (17)	−0.0045 (15)	0.0176 (17)
C1	0.052 (2)	0.076 (3)	0.039 (2)	0.0125 (18)	0.0021 (16)	0.0133 (18)
C2	0.146 (5)	0.079 (3)	0.047 (3)	0.030 (3)	0.012 (3)	0.017 (2)
C3	0.230 (8)	0.074 (3)	0.057 (3)	0.036 (4)	0.021 (4)	0.018 (3)
C4	0.123 (4)	0.081 (3)	0.066 (3)	0.017 (3)	0.006 (3)	0.025 (3)
C5	0.082 (3)	0.098 (4)	0.053 (3)	0.018 (3)	0.012 (2)	0.030 (3)
C6	0.059 (2)	0.091 (3)	0.048 (2)	0.015 (2)	0.0018 (19)	0.018 (2)
C7	0.057 (2)	0.051 (2)	0.049 (2)	0.0156 (17)	−0.0071 (17)	0.0100 (16)
C8	0.062 (3)	0.088 (3)	0.062 (3)	0.026 (2)	0.003 (2)	0.009 (2)
C9	0.058 (3)	0.112 (4)	0.089 (4)	0.024 (3)	0.002 (3)	0.012 (3)
C10	0.070 (3)	0.080 (3)	0.096 (4)	0.025 (2)	−0.029 (3)	−0.001 (3)
C11	0.081 (3)	0.063 (3)	0.065 (3)	0.012 (2)	−0.025 (2)	0.010 (2)
C12	0.065 (3)	0.074 (3)	0.050 (2)	0.007 (2)	−0.0075 (19)	0.015 (2)
C13	0.226 (7)	0.068 (3)	0.026 (2)	0.061 (4)	0.018 (3)	0.0116 (19)
Cl2	0.244 (3)	0.239 (3)	0.0830 (12)	0.035 (2)	−0.0157 (15)	0.0602 (15)
S2	0.0490 (5)	0.0529 (5)	0.0731 (7)	0.0173 (4)	0.0041 (4)	0.0259 (5)
O3	0.0608 (16)	0.0743 (19)	0.095 (2)	0.0364 (14)	0.0125 (15)	0.0398 (16)
O4	0.0641 (17)	0.0545 (17)	0.097 (2)	0.0071 (13)	−0.0088 (16)	0.0213 (15)
N2	0.0595 (19)	0.0517 (19)	0.078 (2)	0.0282 (16)	0.0181 (17)	0.0319 (17)
C14	0.057 (2)	0.055 (2)	0.066 (3)	0.0187 (18)	0.0156 (19)	0.0258 (19)
C15	0.103 (4)	0.063 (3)	0.073 (3)	0.019 (3)	0.007 (3)	0.015 (2)
C16	0.152 (6)	0.096 (4)	0.081 (4)	0.010 (4)	0.001 (4)	0.009 (3)
C17	0.131 (5)	0.131 (6)	0.071 (4)	0.027 (4)	0.003 (4)	0.041 (4)
C18	0.093 (4)	0.103 (4)	0.089 (4)	0.029 (3)	0.011 (3)	0.053 (3)
C19	0.070 (3)	0.063 (3)	0.079 (3)	0.021 (2)	0.007 (2)	0.032 (2)
C20	0.053 (2)	0.0466 (19)	0.046 (2)	0.0183 (16)	−0.0057 (16)	0.0085 (15)
C21	0.062 (2)	0.057 (2)	0.070 (3)	0.0214 (19)	0.001 (2)	0.019 (2)
C22	0.081 (3)	0.057 (2)	0.076 (3)	0.032 (2)	−0.010 (2)	0.008 (2)
C23	0.076 (3)	0.089 (3)	0.070 (3)	0.051 (3)	−0.004 (2)	0.001 (2)
C24	0.063 (3)	0.087 (3)	0.078 (3)	0.032 (2)	0.015 (2)	0.024 (3)
C25	0.056 (2)	0.061 (2)	0.058 (2)	0.0166 (18)	−0.0001 (18)	0.0164 (19)
C26	0.171 (6)	0.045 (3)	0.104 (4)	0.007 (3)	−0.003 (4)	0.010 (3)

### Geometric parameters (Å, °)

**Table:**

Cl1—C4	1.735 (6)	Cl2—C17	1.727 (6)
S1—O1	1.415 (3)	S2—O4	1.417 (3)
S1—O2	1.441 (3)	S2—O3	1.431 (3)
S1—N1	1.611 (4)	S2—N2	1.604 (3)
S1—C1	1.754 (4)	S2—C14	1.756 (4)
N1—C7	1.419 (5)	N2—C20	1.419 (4)
N1—H1N	0.91 (5)	N2—H2N	0.88 (4)
C1—C2	1.382 (6)	C14—C19	1.385 (5)
C1—C6	1.395 (5)	C14—C15	1.389 (6)
C2—C3	1.380 (7)	C15—C16	1.383 (7)
C2—C13	1.571 (6)	C15—C26	1.529 (7)
C3—C4	1.390 (7)	C16—C17	1.378 (8)
C3—H3	0.9300	C16—H16	0.9300
C4—C5	1.340 (7)	C17—C18	1.360 (8)
C5—C6	1.367 (6)	C18—C19	1.366 (7)
C5—H5	0.9300	C18—H18	0.9300
C6—H6	0.9300	C19—H19	0.9300
C7—C8	1.372 (5)	C20—C25	1.367 (5)
C7—C12	1.380 (5)	C20—C21	1.381 (5)
C8—C9	1.388 (6)	C21—C22	1.389 (5)
C8—H8	0.9300	C21—H21	0.9300
C9—C10	1.372 (7)	C22—C23	1.379 (6)
C9—H9	0.9300	C22—H22	0.9300
C10—C11	1.353 (7)	C23—C24	1.361 (6)
C10—H10	0.9300	C23—H23	0.9300
C11—C12	1.377 (6)	C24—C25	1.378 (5)
C11—H11	0.9300	C24—H24	0.9300
C12—H12	0.9300	C25—H25	0.9300
C13—H13A	0.9600	C26—H26A	0.9600
C13—H13B	0.9600	C26—H26B	0.9600
C13—H13C	0.9600	C26—H26C	0.9600
			
O1—S1—O2	118.88 (18)	O4—S2—O3	118.30 (18)
O1—S1—N1	110.6 (2)	O4—S2—N2	110.14 (19)
O2—S1—N1	103.80 (17)	O3—S2—N2	104.50 (17)
O1—S1—C1	106.88 (19)	O4—S2—C14	107.02 (18)
O2—S1—C1	109.17 (19)	O3—S2—C14	109.55 (19)
N1—S1—C1	107.02 (18)	N2—S2—C14	106.81 (18)
C7—N1—S1	126.6 (3)	C20—N2—S2	128.7 (3)
C7—N1—H1N	113 (3)	C20—N2—H2N	116 (3)
S1—N1—H1N	119 (3)	S2—N2—H2N	115 (3)
C2—C1—C6	120.1 (4)	C19—C14—C15	120.7 (4)
C2—C1—S1	122.2 (3)	C19—C14—S2	117.1 (3)
C6—C1—S1	117.7 (3)	C15—C14—S2	122.3 (3)
C3—C2—C1	117.7 (4)	C16—C15—C14	117.3 (5)
C3—C2—C13	118.5 (4)	C16—C15—C26	119.1 (5)
C1—C2—C13	123.8 (4)	C14—C15—C26	123.6 (5)
C2—C3—C4	120.8 (5)	C17—C16—C15	121.0 (6)
C2—C3—H3	119.6	C17—C16—H16	119.5
C4—C3—H3	119.6	C15—C16—H16	119.5
C5—C4—C3	121.4 (5)	C18—C17—C16	121.4 (6)
C5—C4—Cl1	120.5 (4)	C18—C17—Cl2	119.5 (5)
C3—C4—Cl1	118.0 (4)	C16—C17—Cl2	119.1 (6)
C4—C5—C6	118.7 (4)	C17—C18—C19	118.4 (5)
C4—C5—H5	120.6	C17—C18—H18	120.8
C6—C5—H5	120.6	C19—C18—H18	120.8
C5—C6—C1	121.2 (4)	C18—C19—C14	121.1 (5)
C5—C6—H6	119.4	C18—C19—H19	119.4
C1—C6—H6	119.4	C14—C19—H19	119.4
C8—C7—C12	119.8 (4)	C25—C20—C21	120.2 (3)
C8—C7—N1	116.9 (4)	C25—C20—N2	116.9 (3)
C12—C7—N1	123.3 (4)	C21—C20—N2	123.0 (3)
C7—C8—C9	119.3 (4)	C20—C21—C22	119.3 (4)
C7—C8—H8	120.4	C20—C21—H21	120.4
C9—C8—H8	120.4	C22—C21—H21	120.4
C10—C9—C8	120.7 (5)	C23—C22—C21	120.1 (4)
C10—C9—H9	119.7	C23—C22—H22	120.0
C8—C9—H9	119.7	C21—C22—H22	120.0
C11—C10—C9	119.5 (4)	C24—C23—C22	119.9 (4)
C11—C10—H10	120.2	C24—C23—H23	120.1
C9—C10—H10	120.2	C22—C23—H23	120.1
C10—C11—C12	120.9 (4)	C23—C24—C25	120.5 (4)
C10—C11—H11	119.6	C23—C24—H24	119.7
C12—C11—H11	119.6	C25—C24—H24	119.7
C11—C12—C7	119.9 (4)	C20—C25—C24	120.1 (4)
C11—C12—H12	120.1	C20—C25—H25	119.9
C7—C12—H12	120.1	C24—C25—H25	119.9
C2—C13—H13A	109.5	C15—C26—H26A	109.5
C2—C13—H13B	109.5	C15—C26—H26B	109.5
H13A—C13—H13B	109.5	H26A—C26—H26B	109.5
C2—C13—H13C	109.5	C15—C26—H26C	109.5
H13A—C13—H13C	109.5	H26A—C26—H26C	109.5
H13B—C13—H13C	109.5	H26B—C26—H26C	109.5
			
O1—S1—N1—C7	54.0 (4)	O4—S2—N2—C20	−46.3 (4)
O2—S1—N1—C7	−177.4 (3)	O3—S2—N2—C20	−174.3 (3)
C1—S1—N1—C7	−62.0 (4)	C14—S2—N2—C20	69.6 (4)
O1—S1—C1—C2	174.7 (4)	O4—S2—C14—C19	6.9 (4)
O2—S1—C1—C2	44.9 (5)	O3—S2—C14—C19	136.3 (3)
N1—S1—C1—C2	−66.8 (4)	N2—S2—C14—C19	−111.1 (3)
O1—S1—C1—C6	−4.2 (4)	O4—S2—C14—C15	−173.1 (4)
O2—S1—C1—C6	−134.0 (3)	O3—S2—C14—C15	−43.7 (4)
N1—S1—C1—C6	114.2 (3)	N2—S2—C14—C15	68.9 (4)
C6—C1—C2—C3	−1.2 (8)	C19—C14—C15—C16	−0.3 (7)
S1—C1—C2—C3	179.9 (5)	S2—C14—C15—C16	179.7 (4)
C6—C1—C2—C13	−179.3 (5)	C19—C14—C15—C26	179.9 (5)
S1—C1—C2—C13	1.7 (8)	S2—C14—C15—C26	−0.1 (7)
C1—C2—C3—C4	2.1 (10)	C14—C15—C16—C17	0.5 (9)
C13—C2—C3—C4	−179.7 (6)	C26—C15—C16—C17	−179.6 (6)
C2—C3—C4—C5	−2.0 (11)	C15—C16—C17—C18	−1.3 (11)
C2—C3—C4—Cl1	177.8 (6)	C15—C16—C17—Cl2	−178.9 (5)
C3—C4—C5—C6	1.0 (9)	C16—C17—C18—C19	1.8 (10)
Cl1—C4—C5—C6	−178.9 (4)	Cl2—C17—C18—C19	179.4 (4)
C4—C5—C6—C1	−0.1 (7)	C17—C18—C19—C14	−1.5 (8)
C2—C1—C6—C5	0.3 (7)	C15—C14—C19—C18	0.8 (7)
S1—C1—C6—C5	179.2 (3)	S2—C14—C19—C18	−179.2 (4)
S1—N1—C7—C8	163.5 (3)	S2—N2—C20—C25	−176.1 (3)
S1—N1—C7—C12	−17.8 (6)	S2—N2—C20—C21	5.9 (6)
C12—C7—C8—C9	0.5 (6)	C25—C20—C21—C22	−0.5 (6)
N1—C7—C8—C9	179.2 (4)	N2—C20—C21—C22	177.4 (4)
C7—C8—C9—C10	0.1 (7)	C20—C21—C22—C23	0.7 (6)
C8—C9—C10—C11	−0.2 (8)	C21—C22—C23—C24	−0.2 (7)
C9—C10—C11—C12	−0.3 (7)	C22—C23—C24—C25	−0.5 (7)
C10—C11—C12—C7	0.9 (7)	C21—C20—C25—C24	−0.2 (6)
C8—C7—C12—C11	−1.0 (6)	N2—C20—C25—C24	−178.2 (4)
N1—C7—C12—C11	−179.7 (4)	C23—C24—C25—C20	0.7 (7)

### Hydrogen-bond geometry (Å, °)

**Table:**

*D*—H···*A*	*D*—H	H···*A*	*D*···*A*	*D*—H···*A*
N1—H1N···O2^i^	0.91 (5)	2.02 (5)	2.922 (4)	175 (4)
N2—H2N···O3^ii^	0.88 (4)	2.03 (5)	2.906 (4)	173 (4)

Symmetry codes: (i) −*x*+1, −*y*+2, −*z*; (ii) −*x*, −*y*+1, −*z*+1.

## References

[bb1] Enraf–Nonius (1996). *CAD-4-PC* Enraf–Nonius, Delft, The Netherlands.

[bb2] Gelbrich, T., Hursthouse, M. B. & Threlfall, T. L. (2007). *Acta Cryst.* B**63**, 621–632. 10.1107/S010876810701395X 17641433

[bb3] Gowda, B. T., Foro, S., Babitha, K. S. & Fuess, H. (2008*a*). *Acta Cryst.* E**64**, o1692. 10.1107/S1600536808024562 PMC2960504 21201681

[bb4] Gowda, B. T., Foro, S., Babitha, K. S. & Fuess, H. (2008*b*). *Acta Cryst.* E**64**, o1825. 10.1107/S1600536808026895 PMC2960722 21201800

[bb5] Gowda, B. T., Foro, S., Nirmala, P. G., Babitha, K. S. & Fuess, H. (2009). *Acta Cryst.* E**65** Submitted. 10.1107/S160053680900573X PMC2968648 21582231

[bb6] North, A. C. T., Phillips, D. C. & Mathews, F. S. (1968). *Acta Cryst.* A**24**, 351–359.

[bb7] Perlovich, G. L., Tkachev, V. V., Schaper, K.-J. & Raevsky, O. A. (2006). *Acta Cryst.* E**62**, o780–o782.

[bb8] Sheldrick, G. M. (2008). *Acta Cryst.* A**64**, 112–122. 10.1107/S0108767307043930 18156677

[bb9] Spek, A. L. (2003). *J. Appl. Cryst.* **36**, 7–13.

[bb10] Stoe & Cie (1987). *REDU4* Stoe & Cie GmbH, Darmstadt, Germany.

